# Lack of genotoxicity of iron oxide maghemite (γ-Fe_2_O_3_) and magnetite (Fe_3_O_4_) nanoparticles to *Oreochromis niloticus* after acute exposures

**DOI:** 10.1590/1678-4685-GMB2023-0330

**Published:** 2024-09-20

**Authors:** Maria Luiza Fascineli, Paolin Rocio Cáceres-Vélez, Willie Oliveira Pinheiro, Sacha Braun Chaves, Marcelo Henrique Sousa, Wilson Sacchi Peternella, Frederico Hillesheim Horst, Michele de Castro Fernandes, Wania Guimarães, Ricardo Bentes Azevedo, Cesar Koppe Grisolia

**Affiliations:** 1 Universidade de Brasília, Instituto de Ciências Biológicas, Departamento de Genética e Morfologia, Brasília, DF, Brazil.; 2 Universidade Federal da Paraíba, Centro de Ciências da Saúde, Departamento de Morfologia, João Pessoa, PB, Brazil.; 3 Universidade de Brasília, Faculdade de Ceilândia, Brasília, DF, Brazil.; 4 Universidade Federal de Rondônia, Departamento de Química, Porto Velho, RO, Brazil.

**Keywords:** Magnetite, maghemite, tilapia fish, nanotoxicology, nanoparticles

## Abstract

Iron oxide nanoparticles (FeO-NPs) are widely used in scientific and technological fields. Environmental concerns have been raised about residual FeO-NPs levels as their toxicity and bioaccumulative potential are not well understood. *Oreochromis niloticus* were exposed to nanoparticles of γ-Fe_2_O_3_ and Fe_3_O_4._ Micro-CT 3D image and grayscale graphic assessments revealed the accumulation of radiopaque material in the digestive tract of fish exposed to FeO-NPs. Histological analysis showed the presence of such NPs in the hepatopancreas, gills, kidneys, and muscles. No genotoxicity occurred, through micronucleus test and comet assay in peripheral erythrocytes. Body clearance was confirmed by iron-content reduction in organisms exposed to FeO-NPs after recovery period. No tissue injuries were observed in the exposed animals which may be attributed to the absence or low toxicity of iron oxide nanoparticles under the study conditions. *O. niloticus* showed tolerance to sublethal exposures to FeO-NPs.

## Introduction

Nanotechnology is a major innovative scientific and economic growth area (Farré et al., 2009) comprising the study, manipulation, construction materials, substances, devices and objects that have exhibit specific properties relating to nanoscale (Ostiguy et al., 2009). The exponential growth of nanotechnology can introduce a considerable amount of new nanomaterials (NMs) into the environment (Oberdörster et al., 2005), that may affect aquatic/terrestrial organisms and have a detrimental impact on human health (Nowack and Bucheli, 2007). Once released into the environment, engineered nanomaterials can aggregate to some degree, possibly associating with suspended solids and sediment. They may even be accumulated by organisms after entering drinking water sources and food materials (Boxall et al., 2007), the environmental and/or health consequences of which are not fully understood. 

Among the commercially available nanomaterials, one can include the metal oxides, *e.g*. TiO_2_, aluminum oxides and iron oxides. Iron oxide nanoparticles (FeO-NPs) have wide applications in industry, the environment and biomedicine. These applications are correlated with specific size, shape, surface characteristics, and especially magnetic properties (Teja and Koh, 2009), as observed in maghemite (γ-Fe_2_O_3_) and magnetite (Fe_3_O_4_). 

Increased commercial use of iron oxide nanoparticles could result in their release into the environment and aquatic ecosystems in large quantities posing risks to aquatic and/or terrestrial organisms (Ates et al., 2016). Research into the associated ecological impacts and health risks is limited because FeO-NPs are generally considered to present little or no toxicity (Zhang et al., 2015; Ali et al., 2016). However, there is scientific literature documenting the production of reactive oxygen species (ROS) (Patil et al., 2015) after exposure to FeO-NPs with the ability to stimulate cell membrane lipid peroxidation, promoting toxic effects (Valdiglesias et al., 2015). Moreover, FeO-NPs could serve as significant carriers of toxic environmental chemicals and increase exposure to adsorbed pollutants (Zhang *et al*., 2015).

The ability to recognize potentially harmful *in vivo* effects after exposure to metallic nanoparticles, including FeO-NPs, is indispensable given their broad use. In general, metal pollution has constituted an environmental issue in many developed and developing countries for decades. Therefore, there remains a substantial need to not only understand the bioaccumulation and toxicity of metals in aquatic organisms (Wang and Rainbow, 2008), but also to better understand these effects in relation to NMs and identify any possible toxicity resulting from exposure/accumulation. 

In general, both acute and chronic toxicity tests are used to verify material safety and identify any lethal/sublethal effects resulting from its aquatic exposure. In acute toxicity tests, fish are usually exposed to the test item for 96 h. Clinical and behavioral abnormalities, morbidity and mortality are recorded, together with the determination of the lethal concentration, based on recommended OECD protocol for fish acute toxicity test number 203 (OECD, 2019). Although chronic studies (>7 days) are more realistic about the concentrations of chemicals found in the environment, acute aquatic toxicity testing remains a basic requirement for chemical registration in most countries (Elijah et al., 2015). Acute toxicity represents a key property in defining the hazard of large quantities of a substance in cases of accidents or major spillages (GHS, 2019). 

An environmental risk assessment is required to provide information about exposure levels and the hazard(s) that a chemical poses to organisms, with the same assessment paradigm used for NM appraisal (Brink et al., 2019). In addition, some authors suggest that evaluations of uptake, biodistribution, and clearance are useful endpoints for characterizing exposure to NMs and their interaction with biota (Pomeren et al., 2017). Brink *et al.* (2019) considered that the evaluation and quantification of absorption and elimination by organisms are essential in NM environmental risk assessments. However, there are still many uncertainties about how to analyze the toxicological potential of NMs.

Therefore, in this study we examined the intake, uptake, accumulation, and the elimination of FeO-NPs (γ-Fe_2_O_3_ and Fe_3_O_4_), together with genotoxicity evaluations in a tilapia fish (*Oreochromis niloticus*) after acute exposures. Recovery test was also carried out with the understanding how an aquatic organism manages the environmental risks of nanomaterials.

## Material and Methods

### Synthesis and characterization of iron oxide nanoparticles

FeO-NPs were prepared in accordance with the procedure described by Peternele et al. (2014). Briefly, magnetite (Fe_3_O_4_) nanoparticles were synthesized by co-precipitation of Fe^3+^ and Fe^2+^ ions in an alkaline solution. The resulting precipitate was washed until neutral pH and dried at 40 °C for 24 h. Maghemite (γ-Fe_2_O_3_) nanoparticles were obtained by oxidation of the as-prepared magnetite powder for 3 h at 250 °C, in air atmosphere. 

Crystallographic analysis of the samples was performed using the X-ray powder diffraction (XRD) method. Diffraction patterns (20 degrees) were recorded by a Bruker AXL Mod. D8 diffractometer equipped with a copper cathode (Cu Kα_1_ 1,5418 Aº) and Ni filter, operating at 40 kV and a current of 20 mA. A continuous scan of 2 deg/min mode was used to collect 20 data from 20 to 70 degrees. An X-ray diffractogram was plotted with the aid of the Microcal Origin 6.0 software (Microcal Software Inc., Northampton, MA, USA). The full width at half maximum of the (311) reflection was used for particle size determination together with the Scherrer equation (Morais et al., 2001). FeO-NPs morphology were evaluated by Transmission Electron Microscopy (TEM) using a JEM-2100F microscope (Tokyo, Japan). For Principal Group the concentration of 25 and 50 mg/L were also evaluated for some parameters.

### *In vivo* studies

Young adult *Oreochromis niloticus* (Tilapia) fish were obtained from a local fish farm (NUPISC/ SEAGRI-DF, Brasilia, Brazil) where breeding and sanitary conditions were constantly monitored and controlled. Fish of approximately 9±2 cm in length were used in the Principal Group (PG), in which fishes were exposed to FeO-NPs for 24 or 96 h (exposure phase). Animals were subsequently euthanized. The Satellite Group (SG) animals were exposed to FeO-NPs for 96 h, after which time the water was replaced with fresh water without NMs (recovery time) for a further 96 or 192 h. Both the PG and SG consisted of 3 subgroups (control, Fe_3_O_4_ and γ-Fe_2_O_3_) with a maximum of 8 fish for each time period (24, 96, 192 or 288 h) and were exposed to FeO-NPs at 0 mg/L (control group), 100 mg/L (Fe_3_O_4_) or 100 mg/L (γ-Fe_2_O_3_). 

Throughout the experimental phase, all fish were housed in aquariums with: photoperiods of 10 h light/14 h dark, pH 7.5 ± 0.5, constant aeration, and 26 ±1 °C, with water changed every 96 h. The animals received commercial food once a day in the morning, except during the 96-h exposure phase or 192 and 288 h post-exposure phase. Physicochemical parameters, such as dissolved oxygen, nitrites and ammonia, were measured pre- and post-exposure using commercial kits (Labcon®) and conductivity (PHTEKCD203). All parameters remained within the value ranges proposed by the OECD guideline 203 (2019).

### Genotoxic evaluations - micronucleus test and comet assay

Peripheral erythrocytes of *Oreochromis niloticus* were used to detect genotoxic effects caused by exposure to γ-Fe_2_O_3_ or Fe_3_O_4_ after 96 hours of exposure, followed or not by a period without exposure, to verify possible recovery or late effects from tested nanoparticles. Peripheral erythrocytes were collected and evaluated for the formation of micronuclei and nuclear abnormalities, as well as for comet assay after exposures at 0.0, 25.0, 50.0 and 100.0 mg/L of both NPs. In the satellite groups, exposures occurred only at 100.0 mg/L at 192 and 288 h, to follow the recovery group. 

For micronucleus, nuclear abnormalities and comet assay, peripheral blood samples were homogenized in 1 mL of fetal bovine calf and low melt agarose respectively. From this sample, 0.5 mL were used for smear in the micronucleus (MN) and nuclear abnormalities (NA) study. 2000 erythrocytes were scored for MN and 2000 for NA at 1000 magnification, and they were evaluated under a blind code. Erythrocytes were also scored to classify nuclear abnormalities such as BB - blebbed, LB - lobed, NT - notched, BN - binucleated and NB - nuclear bud (Al-Sabti and Metcalfe, 1995). For comet assay - alkaline test blood samples were homogenized in 100 µL of low melt agarose at 0.5% at 37 °C, then these samples were distributed on microscope slides, and covered with a coverslip of 60 mm. The slides were kept in lyse solution for 1 h at 4 °C, and electrophoresis occurred at 0.85 V/cm and 4 °C for 15 min. The cell (nucleoid) analysis for comet classifications followed the protocol developed by Singh et al. (1988), with modifications. One hundred nucleoids per fish were analyzed (blind analysis) and classified based on tail length. During the exposure and post-exposure phases, mortality, clinical signs, and behavioral changes were recorded, twice a day. After exposure, or the post-exposure phase, animals were euthanized with 1% benzocaine hydrochloride in the water. Fish and tissue fragments were subsequently processed. 

### High-resolution X-ray microtomography (micro-CT)

Three animals from each group were euthanized 24, 96 (PG) and 192 h (SG), placed in Davidson’s fixative solution for 24 h and stored in 70% alcohol. Three-dimensional computerized microtomography images of tilapia fish exposed (or not) to FeO-NPs were obtained to evaluate the fate of metals in the body.

Tilapia fish were scanned in a Skyscan 1076 MicroCT (Skyscan, Kontich, Belgium) at 50 kV, 141 µA, Al 0.5 mm filter and 12.56 pixel size. Reconstruction was performed using NRecon software (Skyscan, Kontich, Belgium), applying smoothing, beam-hardening and ring-artifact correction at 01, 10 and 07 level, respectively. Grayscale range was set from 0.2386 to 0.103696 HU. The reconstructed MicroCT files were used to analyze the samples and to create volume renderings of the region of interest, using the CT-Analyzer software (Skyscan, Kontich, Belgium).

### Inductively coupled plasma optical emission spectrometry (ICP-OES)

Quantitative analysis of FeO-NP biodistribution, by means of the dosage of iron content in biological material, was performed with inductively coupled plasma optical emission spectrometry (ICP-OES) using an Optima™ 8000 ICP-OES Spectrometer. Sample preparation involved a tissue fragment of each fish (gill, hepatopancreas, kidney and muscle) and collection of an aliquot of the blood of 5 fish which were weighed and frozen at the end of the exposure (96 h) or recovery time (192 or 288 h). These samples were subsequently dried using an Integrated SpeedVac^®^ System, SAVANT SPD2010 (Thermo Electron Corporation, Milford, MA, US). The dried samples were submitted to acid digestion using nitric acid (70% HNO_3_) for 48 h at RT, as proposed in the literature (Ashoka et al., 2009; Sousa et al., 2011). After diluting samples with ultrapure water, the iron content was measured by ICP-OES and expressed as mg Fe/kg of fresh tissue for all tissues.

### Perl’s Prussian Blue Staining

Gill, hepatopancreas and intestine tissue fragments of the same 5 fish utilized in ICP-OES were fixed, dehydrated and embedded in paraffin. Samples were cut with a microtome (LEICA RM2235), stained with Perl’s Prussian Blue, and analyzed by optical microscopy (ZEISS Axioskop 2-HAL 100), to detect the presence of iron.

### Statistical analyses

Differences between control and treated groups for quantitative data were performed with parametric or non-parametric tests according to normality distribution, e.g., ANOVA (F) or Kruskal-Wallis (H) tests, respectively, followed or not by *post-hoc* analysis (Dunnett’s or Dunn’s Methods). Differences between treated groups for different NPs at the same time were analyzed with the *t*-Test or Mann-Whitney test according to normality distribution. Qualitative data were assessed using the Chi-square Test. Analyses were performed by the IBM SPSS Statistics for Windows, Version 20.0 (2011) program.

### Ethics

The project was approved by the Ethics Committee of the University of Brasilia (Protocol 79/2017).

## Results

### Nanomaterial characterization

**Figure 1 -:**
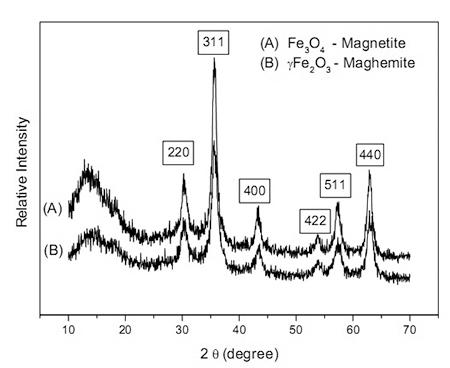
XRD patterns of the magnetite (Fe_3_O_4_) nanoparticles by co-precipitation (A), and maghemite (γ-Fe_2_O_3_) nanoparticles (B).

**Figure 2 -:**
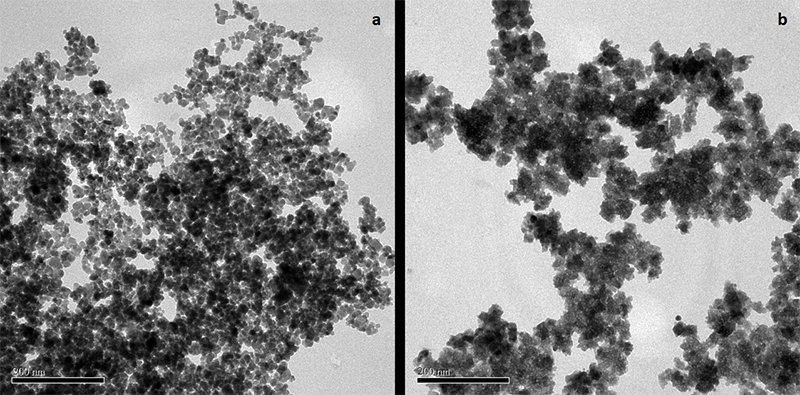
Transmission Electron Microscopy: a) maghemite (γ-Fe_2_O_3_) and b) magnetite (Fe_3_O_4_).

In the present study, nanoparticles were characterized by XRD (Figure 1) and TEM (Figure 2). After coprecipitation of iron salts, a black powder was obtained, indicating formation of the Fe_3_O_4_ phase. The XRD pattern of this sample is shown in Figure 1, with the peaks of the recorded diffractogram readily indexed to the magnetite phase (JCPDS 19-0629). After magnetite oxidation, a reddish-brown precipitate indicative of the conversion of magnetite to maghemite was obtained. The diffracted peaks of this sample were indexed to the γ-Fe_2_O_3_ structure (JCPDS 39-1346) in the pattern shown in Figure 1. The magnetite and maghemite XRD peaks are very analogous; however, as shown in Figure 1, for the oxidized sample, the equivalent XRD peaks are slightly shifted towards the higher angles indicating the predominance of the maghemite phase. More specifically, the quantitative shift of the (511) XRD peak towards 57.5° indicates complete conversion of magnetite into maghemite **(** Silva et al., 2017 **)**. Using Scherrer’s formula to broaden the (311) XRD line, the average crystalline sizes of the Fe_3_O_4_ and γ-Fe_2_O_3_ samples were estimated to be 9.0 and 8.0 nm, respectively.

The TEM images of FeO-NPs in Figure 1 show that the magnetite and maghemite NPs present an almost spherical morphology and are polydisperse in size, as previously observed for this route of synthesis **(** Peternele et al., 2014 **)**.

### Genotoxicity

Mortality, clinical signs and behavioral changes were not observed during the exposure and post-exposure phases. Mutagenic effects (Table 1) were not statistically significant for Principal Group (Np-γ-Fe_2_O_3_ (H =10.546 - p = 0.014 and post test p ˃ 0.05); Np-Fe_3_O_4_ (H = 3.474 - p = 0.324) or Satellite Group (H = 0.692 and p = 0.708) in the recovery time of the 192 h to subgroups exposed to iron oxide nanoparticles compared with control group. However, in the 288 h of the recovery time (H = 8.222 and p = 0.016) the exposure to Np-Fe_3_O_4_ was statistically significant (p ˂ 0.05) for occurrence of micronucleus. 

**Table 1-:** Frequency of micronuclei and nuclear abnormalities in the peripheral blood erythrocytes of *Oriochromis niloticus* exposed to iron oxide nanoparticles.

Principal Group								
	Normal	Micronucleus	Bud	Binucleated	Blebbed	Lobed	Notched	Others
γ-Fe_2_O_3_								
0 mg/L	94.7±3.4	0.0±0.0	0.1±0.1	0.0±0.0	2.1±1.7	0.5±0.4	2.5±1.7	0.1±0.1
25 mg/L	93.7±5.5	0.1±0.1	0.0±0.0	0.0±0.0	3.5±4.1	0.4±0.4	2.2±2.1	0.0±0.0
50 mg/L	98.1±0.8	0.0±0.0	0.0±0.0	0.0±0.0	1.4±0.8	0.2±0.2	**0.4±0.4** ^*^	0.0±0.0
100 mg/L	98.2±0.7	0.0±0.0	0.0±0.0	0.0±0.1	1.1±0.5	0.2±0.1	**0.4±0.2** ^*^	0.0±0.0
Fe_3_O_4_								
0 mg/L	96.9±1.4	0.0±0.0	0.0±0.0	0.0±0.0	2.9±1.3	0.0±0.0	0.2±0.2	0.0±0.0
25 mg/L	98.1±2.0	0.0±0.0	0.1±0.1	0.0±0.0	0.5±0.5	0.2±0.2	1.1±1.3	0.0±0.0
50 mg/L	89.9±9.3	0.0±0.0	**0.5±0.7** ^*^	0.0±0.0	6.4±6.7	**1.1±1.1** ^*^	2.1±1.8	0.0±0.0
100 mg/L	97.5±0.8	0.0±0.0	0.0±0.0	0.0±0.0	1.2±0.7	**0.8±0.3** ^*^	0.5±0.2	0.0±0.0
Satellite Group								
	Normal	Micronucleus	Bud	Binucleated	Blebbed	Lobed	Notched	Others
192 hours								
0 mg/L	97.2±1.5	0.0±0.1	0.0±0.0	0.0±0.0	0.7±0.4	0.8±0.6	1.2±0.7	0.0±0.0
100 mg/L (γ-Fe_2_O_3_)	97.9±1.5	0.0±0.0	0.0±0.0	0.0±0.0	0.9±0.5	0.5±0.5	0.7±0.8	0.0±0.0
100 mg/L (Fe_3_O_4_)	96.8±4.6	0.0±0.0	0.0±0.0	0.0±0.0	2.5±4.8	0.3±0.2	**0.3±0.2** ^*^	0.0±0.1
288 hours								
0 mg/L	93.9±7.5	0.0±0.0	0.0±0.0	0.0±0.0	5.7±7.4	0.2±0.1	0.2±0.3	0.0±0.0
100 mg/L (γ-Fe_2_O_3_)	98.7±0.6	0.0±0.0	0.0±0.0	0.0±0.0	0.6±0.4	0.3±0.2	0.4±0.3	0.0±0.0
100 mg/L (Fe_3_O_4_)	97.7±0.6	**0.1±0.1** ^*^	0.0±0.0	0.0±0.0	0.5±0.4	**0.9±0.4** ^*^	0.8±0.5	0.0±0.0

Two thousand erythrocytes were read per fish (n=6/ subgroup) for analyze of the occurrence of micronuclei or nuclear abnormalities in these cells. The exposed groups (γ-Fe_2_O_3_ or Fe_3_O_4_) were evaluated in relation to the control group of each subgroup; whose statistical differences are represented by an asterisk (*), p˂0.05. Data are represented by mean ± standard deviation (%). Principal Group - 96 hours post exposuse of the test item; Satellite Group - 96 hours post exposure of the test item plus 96 or 192 hours of the additional recovery period (Total - 192 or 288 hours, respectively).

The exposed groups (γ-Fe_2_O_3_ or Fe_3_O_4_) were evaluated in relation to the control group of each subgroup; whose statistical differences are represented by an asterisk (*), p˂0.05. Data are represented by mean ± standard deviation (%). Principal Group - 96 hours post exposure of the test item; Satellite Group - 96 hours post exposure of the test item plus 96 or 192 hours of the additional recovery period (Total - 192 or 288 hours, respectively).

For cytotoxic effects (Table 1), the occurrence of bud nucleus cells in subgroup 50 mg/L after exposure to Np-Fe_3_O_4_ was statistically significant (H = 15.144 - p ˂ 0.05), but this was considered a biological finding without toxicological relevance. Other findings were observed as lobed erythrocyte nuclei after exposure to 50 or 100 mg/L of the Np-Fe_3_O_4_ in Principal Group (H = 11.447 and p = 0.010; post hoc test p ˂ 0.05), as well as in the Satellite Group at 288 h (H = 8.789 and p = 0.012; post hoc test p ˂ 0.05), when compared control groups with Np-Fe_3_O_4_. The exposure to the test item promoted a reduction in the frequency of the notched nucleus cells at Principal Group (Np-γ-Fe_2_O_3_ - 50 and 100 mg/L) and Satellite Group (Np-Fe_3_O_4_ - 100 mg/L) when compared with the control group (H =14.259 and p = 0.003; H = 8.084 and p = 0.018, respectively; post hoc test p ˂ 0.05 - for both). However, the frequency of notched nucleus cells was variable in our groups, in this way, this biological finding was considered without toxicological relevance.

The comet assay showed that there was no statistically significant difference in DNA damage, as represented in Figure 8. The exposure to Np-γ-Fe_2_O_3_ (H = 7.074 - p=0.070) or to Np-Fe_3_O_4_ (F = 0.857 - p = 0.479) for 96 h was not enough to promote DNA fragmentation. No late effects were observed either, during the recovery period (192 and 288 hours), resulting from exposure to Np-γ-Fe_2_O_3_ or Np-Fe_3_O_4_ (F = 2.479 and p = 0.117/ F = 0.623 and p = 0.547, respectively), when compared to control group.

### Micro-CT analysis

**Figure 3 -:**
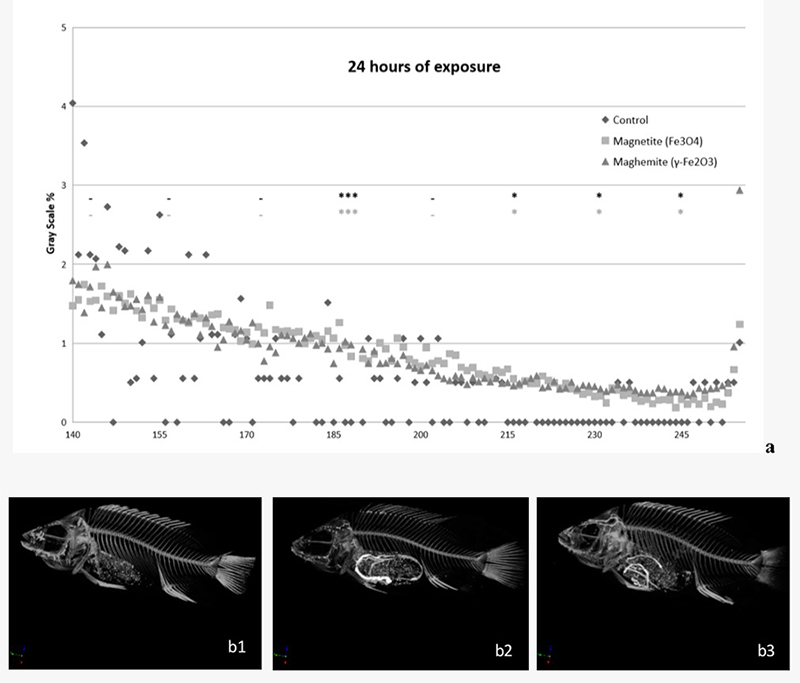
Grayscale graphic of X-ray microtomograph (a) and reconstructed images of tilapia-fishes (b1-3) exposure to γ-Fe_2_O_3_ or Fe_3_O_4_ for 24 h. b1: reconstructed images of control fish in 24 h exposure phase/b2: reconstructed images of fish exposed to γ-Fe_2_O_3_/ b3: reconstructed images of fish exposed to Fe_3_O_4_. The data are represented by the mean of the grayscale values of a given region of interest (ROI), n=3 (per subgroup). Statistical differences are represented with asterisks (*p<0.05; ***p<0.01), in relation to the control group. The absence of significant difference is represented by a dash (- p>0.05). Top row (black) - Fe_3_O_4_ and bottom line (light gray) - γ-Fe_2_O_3_.

**Figure 4 -:**
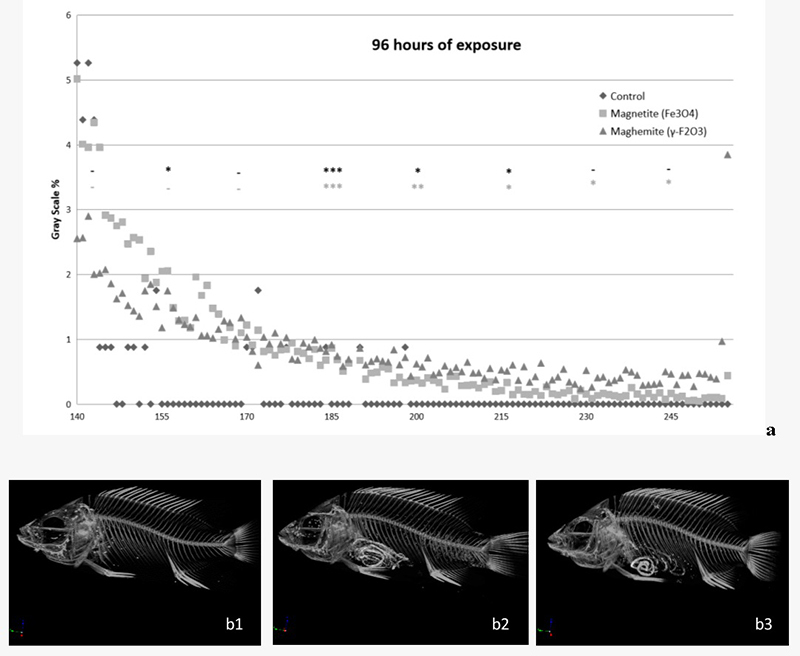
Grayscale graphic of X-ray microtomograph (a) and reconstructed images of tilapia-fish (b1-3) exposed to γ-Fe_2_O_3_ or Fe_3_O_4_ for 96 h. b1:reconstructed images of control fish after 96 h of exposure phase/ b2: reconstructed images of fish exposed to γ-Fe2O3/ b3: reconstructed images of fish exposed to Fe_3_O_4_. The data are represented by the mean of the grayscale values of a given region of interest (ROI), n=3 (per subgroup). Statistical differences are represented with asterisks (*p<0.05; **p<0.01; ***p<0.01), in relation to the control group. Absence of significant difference is represented by a dash (- p>0.05). Top row (black) - Fe_3_O4 and bottom line (light gray) - γ-Fe_2_O_3_.

**Figure 5 -:**
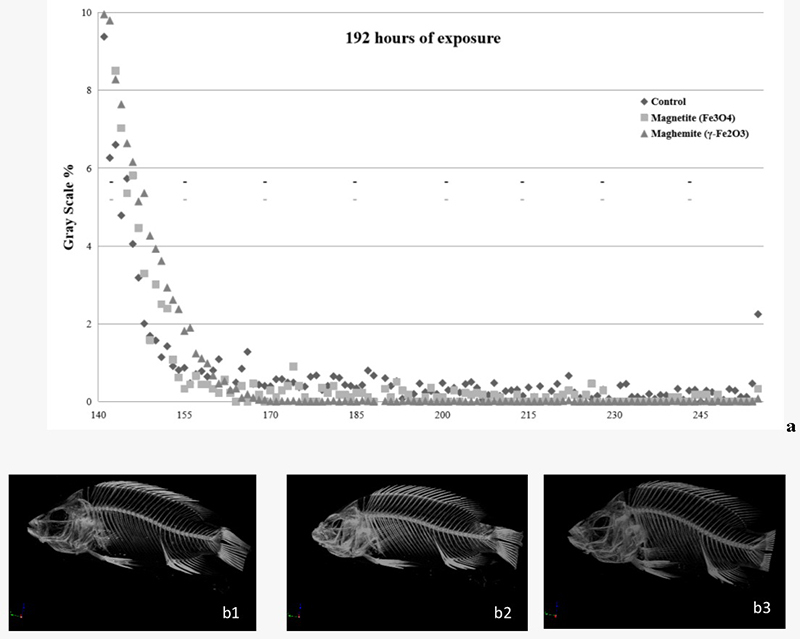
Grayscale graphic of X-ray microtomograph (a) and reconstructed images of tilapia-fish (b1-3) at post-exposure phase (192 h after exposure to γ-Fe_2_O_3_ or Fe_3_O_4_ for 96 h). b1: reconstructed image of control fish in post-exposure phase/ b2: reconstructed image of fish exposed to γ-Fe_2_O_3_ in post-exposure phase/ b3: reconstructed images of fish exposed to Fe_3_O_4_ in post-exposure phase. The data are represented by the mean of the grayscale values of a given region of interest (ROI), n=3 (per subgroup). No statistical difference was observed (- p>0.05). Top row (black) - Fe_3_O_4_ and bottom line (light gray) - γ-Fe_2_O_3_.

In order to construct grayscale graphics, we used the lower and upper threshold values by only selecting voxels within a histogram of all grayscale values of a given region of interest (ROI) that represented differences in the relationship from the control group, as represented in Figures 3a, 4a and 5a. After 24 and 96 h exposure, statistical differences were observed between the exposed groups and the control group, depending on the reading range observed in Figures 3a and 4a, respectively. 

After 24 h of exposure to magnetite or maghemite, the ROI values in the 185 (F = 31.248; p < 0.001 and p < 0.01 for *post-hoc*, respectively) and the 200 (F = 8.169; p < 0.05 for *post-hoc*, to both NPs) ranges were statistically different compared to the control group. 

After 96 h of exposure to magnetite the ROI values in the 155 (F = 7.238; p < 0.05 for *post-hoc*), 170 (F = 15.508; p < 0.01 for *post-hoc*), 185 (F = 40.797; p < 0.001 for *post-hoc*), and 200 (F = 19.825; p < 0.05 for *post-hoc*) ranges also were statistically different to control group; whilst exposure to maghemite for the same time in the 170 (F = 15.508; p < 0.01 for *post-hoc*), 185 (F = 40.797; p < 0.001 for *post-hoc*), 200 (F = 19.825; p < 0.001 for *post-hoc*), 215 (F = 7.019; p < 0.05 for *post-hoc*), 230 (F = 7.125; p < 0.05 for *post-hoc*) and 245 (F = 7.781; p < 0.05 for *post-hoc*) ranges were statistically different compared to the control group. 

**Figure 6 -:**
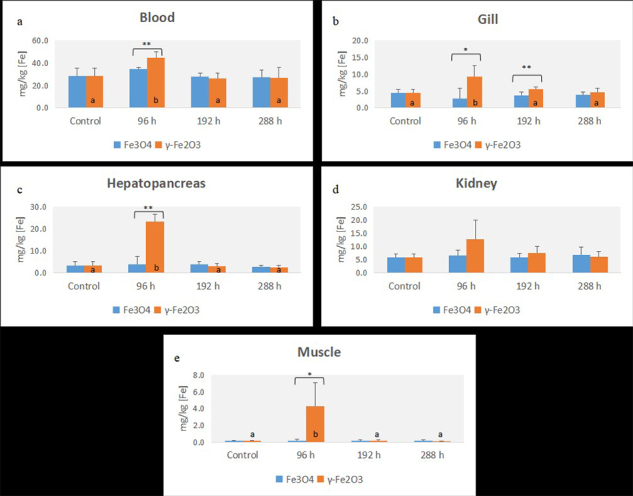
Concentrations of iron content in blood, gill, hepatopancreas, kidney, and muscle (mg/Kg) after 96 h exposure to 0 or 100 mg/L iron oxide nanoparticles (γ-Fe_2_O_3_ or Fe_3_O_4_), at the end of exposure (96 h) or at the end of recovery time (192 h or 288 h). The data are represented by the mean ± standard deviation, n=5 (per subgroup). Different letters (p<0.05) or asterisks (*p<0.05; **p<0.01) show statistical differences to the same nanoparticles at different times compared to the control or between different nanoparticles at the same time, respectively. Genotoxicity of iron nanoparticles

However, no significant differences were observed during recovery time (192 h) when exposure groups to magnetite or maghemite were both compared to control group, as depicted in Figure 6a (ROI-140: F = 0.743; ROI-155: H = 3.377; ROI-170: F = 1.142; ROI-185: F = 0.812; ROI-200: H = 3.396; ROI-215: H = 3.810; ROI-230: H = 1.667; ROI-245: H = 1.774; p > 0.05 for each F or H value). 

Figures 3b and 4b present X-ray reconstructed images, showing FeO-NPs accumulation within the fish digestive system after exposure times of 24h (Figures 3b2 and 3b3) and 96h (Figures 4b2 and 4b3). We did not observe test-item accumulation after 192 h as supported in Figure 5b (b1, b2 and b3) or in the control group (Figures 3b1 and 4b1).

### ICP-OES analysis

The total Fe content was measured by ICP-OES and expressed in mg Fe/kg of gill, hepatopancreas, kidney, muscle, and blood. As observed in Figure 6, fish exposed to maghemite showed an increase in Fe content at 96 h in the blood (6a), gill (6b), hepatopancreas (6c), and muscle (6e) in comparison to the control group (F = 7.691, H = 10.894, H = 9.877, H = 15.234, respectively; p < 0.05 for *post-hoc*, to all tissues) but not for fish exposed to magnetite (H = 6.887, H = 1.627, H = 2.000, H = 6.714, respectively; p > 0.05 for each H value); whilst statistical differences in iron concentration in the kidney (6d) were not observed in relation to the control group for both maghemite (F = 1.435 and p > 0.05) and magnetite (F = 2.011 and p > 0.05). On the other hand, we observed a statistically significant increased iron concentration in the blood (p < 0.01), gills (p < 0.05), hepatopancreas (p < 0.001), and muscle (p < 0.05) in the maghemite group compared to the magnetite group after 96 h; even though these values were not statistically different after 192 or 288 h (p > 0.05). 

These results indicate tissue clearance after the 192 and 288 h recovery times, showing a similar Fe-content for all experimental groups (Figure 6). Regarding iron concentration in the gills, a comparison between groups exposed to NPs (6b) at 192 h showed statistical significance (p < 0.01) for the maghemite group, whose tissue clearance was observed after 288 h (p > 0.05).

### Perl’s Prussian Blue staining

**Figure 7 -:**
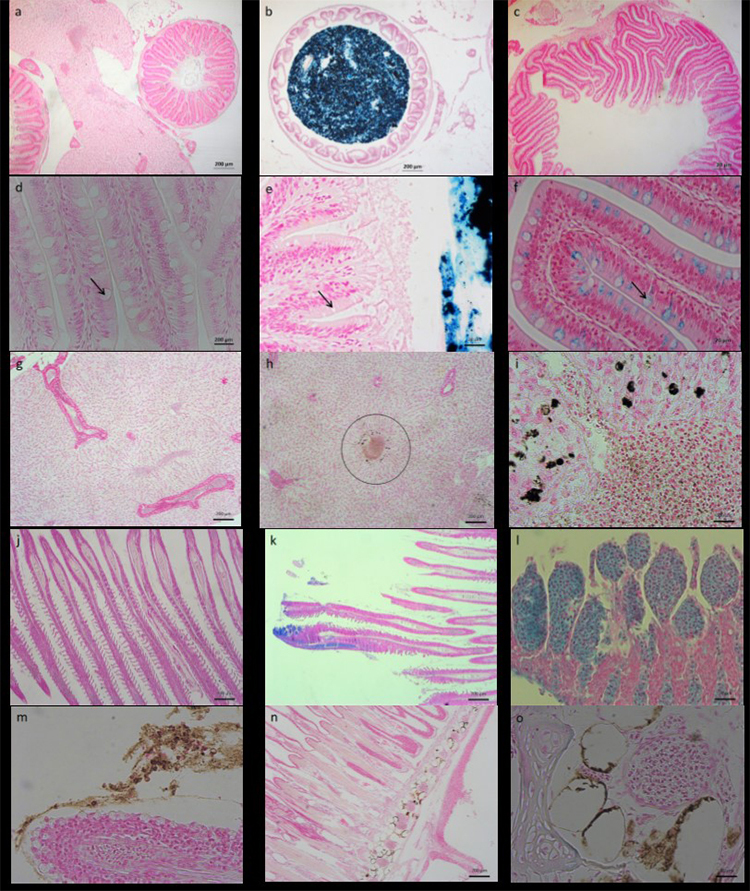
Histological section showing gut and intestinal villi (a-f), hepatopancreas (a, g - i) and gills (j-o) after 96 h of exposure to 0 or 100 mg/L iron oxide nanoparticles (γ-Fe_2_O_3_ or Fe_3_O_4_), at the end of exposure (96 h) or at the end of recovery time (192 h or 288 h), (Perl’s Stain). Control groups are represented in: a, d, g and j. Positive reaction to Perl’s Stain (blue) was possible to observe: in lumen of the intestine after 96 h exposure (b and e γ-Fe_2_O_3_ or Fe_3_O_4_); and in the red cells and lamellae cells of the gills after 96 h exposure (k and l γ-Fe_2_O_3_); in goblet cells (arrows), principally after 288 h of recovery time (c and f γ-Fe_2_O_3_); formalin-heme pigment was observed in hepatopancreas (h - circle; i - detail of h circle) and between lamellae (m) or gill arch (n and o), mainly after exposure to γ-Fe_2_O_3_. This is a representation of the main findings found; the study was performed with n=5 slides/ per tissue/ per fish.

The histological sections in Figure 7 show intestine (7a -f), hepatopancreas (7a, g-i) and gills (7 j-o) stained with Perl’s Prussian Blue. Positive reactions to Perl’s stain (blue) were observed: in the intestinal after 96 h exposure (7b, e γ-Fe_2_O_3_ or Fe_3_O_4_); and in the red cells and lamellae cells of the gills after 96 h exposure (7k, l γ-Fe_2_O_3_); in goblet cells, principally after 288 h recovery (7c, f γ-Fe_2_O_3_); Formalin-heme pigment were observed in hepatopancreas (7h-i) and between lamellae (7m) or gill arch (7n, o), mainly after exposure to γ-Fe_2_O_3_. 

**Figure 8 -:**
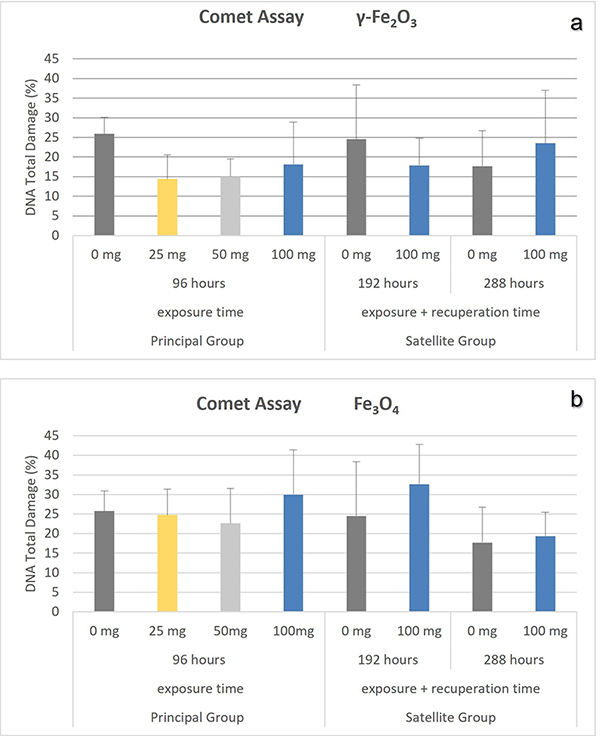
DNA fragmentation (%) of the peripheral blood erythrocyte of *Oreochromis niloticus* exposed after 96 h exposure to iron oxide nanoparticles (γ-Fe_2_O_3_ or Fe_3_O_4_), at the end of exposure (96 h) or at the end of recovery time (192 h or 288 h). The data are represented by the mean ± standard deviation, (n=6/subgroup). No statistical differences were observed after exposure or recovery time when compared to control, p˃0.05.

A positive reaction to Perl’s stain in gills and hepatopancreas was observed in some animals exposed to FeO-NPs; however statistically significant differences were observed in the maghemite group (p < 0.001); this positive and significant reaction was observed in the gills during all experimental period (Control = 6.25%; 96h = 83.3%; 192h = 85.7%; 288h = 57.143%), and, in the hepatopancreas, it was observed only at 96 h (Control = 4.8%; 96 h = 75.0%; 192 h = 0%; 288 h = 0%). 

Besides, a positive reaction to Perl’s stain in the lumen of the intestinal tract of animals exposed to maghemite (p < 0.001) was observed after the 96 h and 192 h exposures (Control = 0%; 96h = 83.3%; 192h = 50%; 288h = 0%); however, the exposure to magnetite caused an increase of the positive reaction (p < 0.001) only after the 96 h exposure (Control = 0%; 96h = 42.9%; 192h = 0%; 288h = 0%). 

Moreover, a statistically significant increase in the positive reaction to Perl’s Stain was observed in the intestinal goblet cells (p < 0.001) of the group exposed to maghemite after 288 h (Control = 9.5%; 96h = 0%; 192h = 0%; 288h = 83.3%), despite the fact it was not statistically different after exposure to magnetite at all of the exposure times (p > 0.05).

## Discussion

Fish are good indicators of metallic contamination in aquatic systems (Andreji et al., 2005; Authman et al., 2015). They are widely used as bio-indicators of metal pollution (Kumar et al., 2011), and could therefore constitute a good metallic NP exposure indicator. Some materials could be accumulating in tissue and pose a health risk to those who frequently consume fish (Andreji *et al*., 2005). In addition, this bioaccumulation could have adverse effects on the exposed organism, e.g. influence homeostasis and reproduction in fish; weaken the immune system and/or induce pathological changes (Authman *et al*., 2015). Among this animal class, *Oreochromis* ssp (Tilapia) is an exotic fish species widely cultivated in different countries for human consumption. These fish are able to accumulate certain environmental substances in their tissues, which could be correlated with human health risks (Jha, 2004; Bawuro et al., 2018). On the other hand, there are few studies in the scientific literature using this species as a model to investigate the consequences of exposure to metallic-based NPs, including metal oxides, or other NMs.

Eighty articles papers were found in scientific databases (e.g. Science direct or PubMed) under ‘Tilapia’ or ‘*Oreochromis’* and (‘nanomaterials’ or ‘nanoparticles’), as observed recently (December 2020). These studies report the adverse effects and/or accumulation of NMs in the fish species *Oreochromis niloticus* or *Oreochromis mossambicus* following chronic or acute exposure. Most of these studies analyzed inorganic NP exposure; e.g.: zinc oxide or metallic zinc NPs (Abdel-Khalek et al., 2015; Alkaladi et al., 2015; 2020; Kaya et al., 2015; 2016; Farsani et al., 2017; Abdelazim et al., 2018; Anjugam et al., 2018; Campos et al., 2019; Suganthi et al., 2019; Mohamed et al., 2020) titanium oxide nanoparticles (Canli et al., 2018; Suganthi *et al.*, 2019;); silver nanoparticles (Govindasamy and Rahuman, 2012; Abu-Elala et al., 2018; Ibrahim, 2020); aluminium oxide nanoparticles (Murali et al., 2017; 2018; Abdel-Khalek *et al*., 2020); gold nanoparticles (Vijayakumar et al., 2017); cadmium nanoparticles or cadmium dioxide nanoparticles (Al-Abdan et al., 2020; Ibrahim *et al*., 2021); copper oxide nanoparticles (Abdel-Khalek *et al*., 2015; Abu-Elala *et al.,* 2018; Shahzad et al., 2018); nickel nanoparticles (Jayaseelan et al., 2014), and iron oxide nanoparticles (Ates et al., 2016; Abdel-Khalek *et al*., 2020). At the same time, these species are widely used in aquaculture as a human source of protein there are few studies using tilapias as test-organism experimentally. So, tilapia-fish could be more explored in terms of their toxicological aspects or a potential source of NMs accumulated after environmental exposure. 

In the present study, an increase in iron accumulation in the gastrointestinal tract of tilapia-fish after acute exposure (96 h) to the main forms of FeO-NPs - magnetite (Fe_3_O_4_) and its oxidized form maghemite (γ-Fe_2_O_3_) - was observed, together with a subsequent decrease in iron content and recovery time. Hu et al. (2012) observed maghemite NP accumulation in the gut of *Ceriodaphnia dubia* after exposure to 5, 25 or 50 mg/L of nano-Fe_2_O_3_, utilizing an optical microscope. In our study, Fe accumulation in the lumen of the digestive tract was detected utilizing X-ray computed microtomography after 24 h and 96 h, and by optical microscopy, when the contents in the lumen of the digestive tract reacted positively to Perl’s reagent after 96 h exposure to FeO-NPs. Additionally, the presence of food in the digestive tract after 24 h exposure did not hinder observation of test-material accumulation in the organism, as observed in the grayscale graphic (Figure 3a) and the reconstructed images (Figures 3b2 and 3b3).

A similar study reported iron accumulation in the gastrointestinal tract of the Medaka larvae fish after exposure to 25−75 nm-sized magnetite NPs, with blue precipitation observed after Perl’s reagent (Chen et al., 2012). In the tilapia fish, accumulation in the lumen of the gastrointestinal tract was observed after 24 and 96 h (Figures 3, 4, 7b and 7e) after exposure to γ-Fe_2_O_3_ or Fe_3_O_4_ NPs, but a reduced or absent accumulation was observed after the 192 h post-exposure phase, the period where fish were placed in clean water after exposure to NPs for 96 h (Figures 5 and 7). In this recovery time, the lumen of the digestive tract showed a reduced or absent reaction to Perl’s and was negative in the micro-CT. A similar result was observed for *Ceriodaphnia dubia* (daphnia) following exposure to 20-40 nm Fe-NPs when this test organism was placed in a clean environment without NPs (Hu et al., 2012).

Even in the absence of a positive Perl´s reaction in the lumen of the digestive tract, a positive Perl’s reaction was observed for goblet cells after the recovery period (Figures 7c and 7f), mainly in the time of 288 h for animals exposed to maghemite. Zhao et al. (2014) demonstrated that goblet cells are a natural pathway for NP excretion in zebrafish and mice. Other authors also verified that metal NPs (*e.g.* silver nanoplates, magnetic Fe_3_O_4_ NPs, gold nanorods, and gold nanoclusters) injected via the tail were excreted into the gut lumen via the secretion of intestinal goblet cells (Liu et al., 2019). From this perspective it was a similar observation to our study, an indication of depuration by goblet cells after oral exposure in a 192 or 288 h recovery time (Figures 7c and 7f).

As important as the excretion via is the route of exposure. There are 2 potential sites for metal uptake in fish, across the intestine (dietary borne) or branchial epithelium (water borne) (Bury and Grosell, 2003). Previously reported results, together with our data, show that the ingestion of FeO-NPs could be the main route of nanometal bioaccumulation in environmentally exposed organisms. We believe that exposure via the digestive tract was correlated with the increased iron concentration in the blood, hepatopancreas and muscle (Figures 6a, c, and e, respectively). An interesting observation is that the increase in iron concentration was only present after 96 h of exposure to maghemite, and not for magnetite NP exposure. In the present study, γ-Fe_2_O_3_ was more readily taken up in acute exposure than Fe_3_O_4,_ through the digestive tract and even by the respiratory tract epithelium. Moreover, this increased iron concentration returned to normal levels during the recovery time, similar to the control group, with the exception of the gills, whose clearance time was longer (Figure 6).

Some authors reported that FeO-NP uptake, distribution, clearance and toxicity depend on NP size and coating (Feng et al., 2018). However, taking into account that the FeO-NPs utilized in this work are bare magnetite and maghemite NPs, similar in terms of size and morphology, our results indicate that the physicochemical characteristics of the NMs, such as composition, metal valence, surface and crystal properties can influence FeO-NP uptake by fish. 

In fact, the structure of magnetite is that of an inverse spinel, with 32 O^2-^ ions regularly organized in a face-centered cubic unit cell with Fe^3+^ and Fe^2+^ ions distributed in octahedral and tetrahedral sites. The structure of maghemite is similar to that of magnetite, however, all or most Fe ions are in a trivalent state. To compensate the oxidation of Fe^2+^, the charge balance is achieved by cation vacancies in the structure (Peternele et al., 2014). Thus, as expected, the XRD data of our samples confirm preservation of the spinel structure during the oxidation process (Figure 1). However, the easy oxidation of divalent iron ions in tetrahedral sites on the Fe_3_O_4_ surface (from the surface to the core) can change the properties of FeO-NPs, such as their surface reactivity (Schwaminger, et al., 2017). Furthermore, Fe^2+^ ions occurring in magnetite are known to have an impact on the interaction of FeO-NPs with biological materials. For instance, maghemite commonly exhibits lower toxic effects than magnetite towards biological organisms (Auffan et al., 2008; Auffan *et al.,* 2009).

Chen et al. (2012) related that exposure to zerovalent iron NPs resulted in gill iron deposition associated with mortality in medaka fish. In our study, a significant increase in iron content was observed in branchiate tissue after exposure to maghemite in comparison with the control (96 h) or with the magnetite group (96 h and 192 h). Some animals presented a positive reaction to Perl’s stain, with altered staining of the erythrocyte cytoplasm or secondary lamellae cells (Figures 7k and l); other fish presented a formalin-heme pigment deposit between lamellae or the gill arch (Figure 7 m-o), without any significant statistical differences or mortality occurrence.

Iron is a vital micronutrient for teleost fish as it is an integral component of proteins involved in cellular respiration and oxygen transfer (Bury and Grosell, 2003; Andreji et al., 2005). However, iron is toxic in excess, so fish need to balance uptake to prevent deficiency/potential toxicity (Bury and Grosell, 2003). The toxicity of iron-based NPs is a function of their properties, tolerance of test organisms and environmental conditions (Lei et al., 2018). In our study, no clinical or behavioral abnormalities were observed in either the principal group or satellite group during or post-exposure to the test item.

A limited concentration of FeO-NPs was utilized (100 mg/L), as proposed by the OECD guideline - 203 (2019). However, this guideline is not specific to evaluate exposure to nanomaterials. No morbidity or mortality was observed in the experimental groups, and the lethal concentration (LC_50_) was higher than the concentration proposed as a test limit for chemicals. These data indicate that FeO-NPs present low toxicity to fish. 

Appropriate physical and chemical characterization of natural and manufactured NPs is fundamental in order to determine their intrinsic properties. Phase purity, particle and cluster size, surface chemistry, solubility, charge and crystallinity are essential to elucidate the homogeneity, stability, reactivity, biodurability and potential application of NPs in different media (Peralta-Videa et al., 2011). The solubility of FeO-NPs in water is extremely low (Brunner et al., 2006). In our study, the γ-Fe_2_O_3_ or Fe_3_O_4_ NPs were insoluble and did not alter the physicochemical parameters of the water. Maghemite is more oxidized, and in a more stable iron oxide phase, than magnetite which could influence toxicity (Singh et al., 2010). In the present study, differences in the oxidation grade of the NPs are not correlated with the occurrence of toxicity, but could be associated with differences in uptake after acute exposure. Metal bioconcentration and bioaccumulation processes depend on: the fish species and their trophic level, sampling location, type of food, type of absorption carried out by the organism, particle size, metal phase (dissolved or particulate) (Voigt et al., 2015), and exposure time. 

Zhang et al. (2015) and Ates et al. (2016) observed the accumulation and distribution of Fe or FeO-NPs in zebrafish (*D. rerio*) and tilapia-fish (*O. niloticus*), after chronic aqueous exposure to nano-Fe_2_O_3_, nano-Fe_3_O_4_, and α-Fe_2_O_3_ and γ-Fe_2_O_3_ NPs, respectively, using ICP-MS. After chronic exposure (60 days), tilapias were transferred to NP-free freshwater resulting in the elimination of ingested NPs within 30 days, except in the hepatopancreas and spleen (Ates *et al.,* 2016). After chronic waterborne exposure (52 days), the accumulated NPs were eliminated efficiently when fish were moved to NP-free water for 24 days post-exposure. According to Fe content analysis of fish excrement during the elimination phase, iron oxide NMs may be adsorbed via the gastrointestinal tract, and stored for more than 12 days (Zhang *et al.,* 2015). 

The genotoxicity of iron-based NPs *in vivo* and *in vitro* from cellular level up to the whole organism are related to ROS-induced oxidative stress, which is the most accepted toxic mechanism (Lei et al., 2018). The comet assay has been successfully used for detection of damages caused by oxidized DNA bases in fish exposed to environmental contaminants. The alkaline version offers increased sensitivity to agents that cause DNA oxidative lesions (Jha, 2008). In this study, comet assay correlates with micronucleus test and nuclear abnormalities, evidencing neither genotoxicity nor cytotoxicity. In conclusion, these results demonstrate that acute exposures to FeO-NPs promotes an increased iron-content in the fish’s body during exposures, which rapidly returns to normal índices throughout the recovery period, with no apparent toxicity. On the other hand, *O. niloticus* can be tolerant to sublethal toxicity of FeO-NPs, developing increased activities of antioxidants enzymes, which were not quantifyed in this study.
